# Homologous Recombination Deficiency in Pancreatic Cancer: A Systematic Review and Prevalence Meta-Analysis

**DOI:** 10.1200/JCO.20.03238

**Published:** 2021-07-01

**Authors:** Raffaella Casolino, Salvatore Paiella, Danila Azzolina, Philip A. Beer, Vincenzo Corbo, Giulia Lorenzoni, Dario Gregori, Talia Golan, Chiara Braconi, Fieke E. M. Froeling, Michele Milella, Aldo Scarpa, Antonio Pea, Giuseppe Malleo, Roberto Salvia, Claudio Bassi, David K. Chang, Andrew V. Biankin

**Affiliations:** ^1^ Wolfson Wohl Cancer Research Centre, Institute of Cancer Sciences, University of Glasgow, Glasgow, United Kingdom; ^2^ Department of Medicine, University and Hospital Trust of Verona, Verona, Italy; ^3^ General and Pancreatic Surgery Unit, Pancreas Institute, University and Hospital Trust of Verona, Verona, Italy; ^4^ Unit of Biostatistics, Epidemiology and Public Health, Department of Cardiac, Thoracic, Vascular Sciences, and Public Health, University of Padova, Padova, Italy; ^5^ Research Support Unit, Department of Translational Medicine, University of Eastern Piedmont, Novara, Italy; ^6^ Sanger Institute, Wellcome Trust Genome Campus, Cambridge, United Kingdom; ^7^ Section of Pathology, Department of Diagnostics and Public Health, University and Hospital Trust of Verona, Verona, Italy; ^8^ ARC-Net Research Centre, University of Verona, Verona, Italy; ^9^ The Oncology Institute, Sheba Medical Center at Tel-Hashomer, Tel Aviv University, Tel Aviv, Israel; ^10^ Beatson West of Scotland Cancer Centre, Glasgow, United Kingdom; ^11^ Section of Oncology, Department of Medicine, University and Hospital Trust of Verona, Verona, Italy; ^12^ West of Scotland Pancreatic Unit, Glasgow Royal Infirmary, Glasgow, United Kingdom; ^13^ Faculty of Medicine, South Western Sydney Clinical School, University of NSW, Liverpool, Australia

## Abstract

To analyze the prevalence of homologous recombination deficiency (HRD) in patients with pancreatic ductal adenocarcinoma (PDAC).

**MATERIALS AND METHODS:**

We conducted a systematic review and meta-analysis of the prevalence of HRD in PDAC from PubMed, Scopus, and Cochrane Library databases, and online cancer genomic data sets. The main outcome was pooled prevalence of somatic and germline mutations in the better characterized HRD genes (*BRCA1*, *BRCA2*, *PALB2*, *ATM*, *ATR*, *CHEK2*, *RAD51*, and the *FANC* genes). The secondary outcomes were prevalence of germline mutations overall, and in sporadic and familial cases; prevalence of germline *BRCA1/2* mutations in Ashkenazi Jewish (AJ); and prevalence of HRD based on other definitions (ie, alterations in other genes, genomic scars, and mutational signatures). Random-effects modeling with the Freeman-Tukey transformation was used for the analyses. PROSPERO registration number: (CRD42020190813).

**RESULTS:**

Sixty studies with 21,842 participants were included in the systematic review and 57 in the meta-analysis. Prevalence of germline and somatic mutations was *BRCA1*: 0.9%, *BRCA2*: 3.5%, *PALB2*: 0.2%, *ATM*: 2.2%, *CHEK2*: 0.3%, *FANC*: 0.5%, *RAD51*: 0.0%, and *ATR*: 0.1%. Prevalence of germline mutations was *BRCA1*: 0.9% (2.4% in AJ), *BRCA2*: 3.8% (8.2% in AJ), *PALB2*: 0.2%, *ATM*: 2%, *CHEK2*: 0.3%, and *FANC*: 0.4%. No significant differences between sporadic and familial cases were identified. HRD prevalence ranged between 14.5%-16.5% through targeted next-generation sequencing and 24%-44% through whole-genome or whole-exome sequencing allowing complementary genomic analysis, including genomic scars and other signatures (surrogate markers of HRD).

**CONCLUSION:**

Surrogate readouts of HRD identify a greater proportion of patients with HRD than analyses limited to gene-level approaches. There is a clear need to harmonize HRD definitions and to validate the optimal biomarker for treatment selection. Universal HRD screening including integrated somatic and germline analysis should be offered to all patients with PDAC.

## INTRODUCTION

Pancreatic ductal adenocarcinoma (PDAC) is the deadliest solid malignancy, with a five-year survival rate of < 10% and an increasing public health burden considering the estimated rise of its incidence and unchanging mortality over the next 20 years.^1-4^ Its biologic aggressiveness is compounded by the limited availability of effective therapies and a lack of prevention strategies.^5,6^ Defects in DNA damage response (DDR) genes causing homologous recombination deficiency (HRD) identify a clinically relevant subgroup of patients with PDAC, with both therapeutic and preventative implications.^7-10^ Accumulating evidence from nonrandomized clinical trials infers HRD as a putative biomarker of therapeutic response for platinum-based chemotherapy in patients with advanced PDAC.^11,12^ Within HRD, germline variants in *BRCA1* and *BRCA2* are associated with improved progression-free survival in patients with platinum-sensitive metastatic PDAC treated with the poly (ADP-ribose) polymerase (PARP) inhibitor (PARPi) olaparib as maintenance therapy.^13^ Interestingly, based on preclinical evidence and phase II nonrandomized clinical trials, additional non-*BRCA* HRD aberrations may predict sensitivity to PARPi^14-17^ with other therapeutic strategies targeting DDR currently under clinical investigation (including immunotherapy, ATM, ATR, and WEE1 inhibitors).^18,19^ In addition, germline pathogenic variants in several HRD genes in PDAC confers cancer susceptibility, with implications for risk assessment and prevention of a broad spectrum of neoplasms in patients and healthy relatives.^20^

**CONTEXT**

**Key Objective**
Homologous recombination deficiency (HRD) identifies a clinically relevant subgroup of patients with pancreatic cancer (pancreatic ductal adenocarcinoma). However, clinically relevant HRD is still poorly defined and variably reported, depending on definitions and assays used. This systematic review and meta-analysis aimed to define the prevalence of HRD in pancreatic ductal adenocarcinoma.
**Knowledge Generated**
Surrogate readouts of HRD (ie, genomic scarring, as well as point mutational and structural variant signatures) can identify a greater proportion of patients with HRD than analyses limited to gene-level approaches. However, a clinically applicable diagnostic is yet to be developed to capture these. The rate of germline mutations in HRD genes is similar in sporadic and familial patients.
**Relevance**
Given the known therapeutic implications of HRD-associated pancreatic cancer (such as sensitivity to platinum analogues and possibly other targeted agents, including poly [ADP-ribose] polymerase inhibitors), identifying patients who fit into this category beyond the use of standard gene panels will be important to refine our approach to precision medicine in this disease.

Despite major efforts, HRD is still challenging to define with reported prevalence in PDAC highly variable, limiting its clinical implementation in routine practice and therapeutic development.^7,11,12,18,21-29^ This uncertainty is primarily because of inconsistencies in HRD measurement and definitions (gene-level tests, genomic scars, signatures, or a combination of these methods); and the difficulties in assessing the contribution of each genomic event.^30,31^ Specifically, a few hundred genes are proposed to be involved in homologous recombination repair, including (but not limited to) *BRCA1/2*, *PALB2*, *ATR*, *ATM*, *CHEK1/2*, *RAD51*, and *FANC* genes, resulting in HRD when germline or somatic inactivation occurs by mutation or epigenetic silencing.^32^ It is possible to analyze a broad range of these genes in a single test through next-generation sequencing (NGS) technologies. However, there is no accepted consensus on which genes and genomic regions should be included using sequencing methods that can be applied in routine care to maximize the probability of finding clinically meaningful HRD. The inconsistency of genes included in NGS-based HRD panels, and the interpretation of the functional impact of mutations, result in high variability in prevalence estimates of HRD in PDAC.^11,12,18,25,28,29^ Moreover, recent largescale whole-exome sequencing (WES) and whole-genome sequencing (WGS) analyses suggest that HRD likely extends beyond point mutations in core genes, implying other molecular mechanisms, which are yet to be elucidated.^7,21,24,33-36^

Here, we present a systematic review of the current literature on HRD in PDAC and perform a prevalence meta-analysis of the better-characterized HRD genes with known or potential clinical utility. Particular focus was given to germline variants, both in sporadic and familial cases, to assess the contribution of HRD genes to cancer susceptibility and potential intervention.

## MATERIALS AND METHODS

### Search Strategy, Selection, and Inclusion Criteria

The study protocol and data extraction for the systematic review and meta-analysis was designed according to Preferred Reporting Items for Systematic reviews and Meta-Analyses guidelines.^37^ The research protocol was registered at the International Prospective Register of Systematic Reviews (PROSPERO^38^ number: CRD42020190813). PubMed, Scopus, and the Cochrane Library databases, and online cancer genomic data sets were queried for articles reporting the prevalence of HRD in PDAC, published from database inception to February 28, 2020. Specific HRD genes were selected after an exhaustive review of the literature and pragmatic considerations based on which genes were studied and reported in the literature and the likely clinical utility (most frequently altered; better characterized; known role in PDAC susceptibility; used as biomarkers in clinical trials): *BRCA1*, *BRCA2*, *PALB2*, *ATR*, *ATM*, *CHEK2*, and *RAD51* (including -*B*, *C*, *D*), and the Fanconi-Anemia (*FANC*) genes (at least one of the following: *FANC*-*A*, *B*, *C*, *D1*, *D2*, *E*, *F*, *G*, and *M*). Studies reporting other definitions of HRD (ie, mutations in other genes, genomic scars, signatures, and structural variation patterns) were also considered for inclusion in the systematic review but not for the pooled prevalence meta-analysis.

The search protocol was updated on May 19, 2020, after the Food and Drug Administration approval of olaparib for patients with HRD metastatic castration-resistant prostate cancer, defined according to germline or somatic mutations in the following 15 genes: *BRCA1/2*, *ATM*, *BARD1*, *BRIP1*, *CDK12*, *CHEK1/2*, *FANCL*, *PALB2*, *PPP2R2A*, *RAD51 B/C/D*, or *RAD54L*.^39^ As the data extraction was terminated at that time, only online cancer genomic data sets were queried to investigate the prevalence of these 15 genes in PDAC.

Titles and abstracts of all identified articles and publicly available data sets were independently screened by two authors (R.C. and S.P.). Articles were included if the study cohort was composed of at least 20 patients, regardless of study kind and design, sequencing methodology, DNA source, type of mutation, or ethnicity of the study cohorts. Each author worked blindly from the other, and each selected manuscript was double-checked by the other. Discrepancies were resolved through consensus by four authors (R.C., S.P., D.K.C., and V.C.). The possibility of overlapping populations was considered. The von Elm patterns of duplication were adopted.^40^ Further details on search strategy, selection, and data extraction are presented in the Data Supplement (online only).

### Outcomes of Interest and Definitions

The main outcome measure was the pooled prevalence of germline and somatic mutations in each HRD gene listed above. Secondary outcomes included the pooled prevalence of germline mutations overall, and individually in familial and sporadic PDAC; the pooled prevalence of germline *BRCA1/2* mutations in patients with Ashkenazi Jewish (AJ) ancestry; and the prevalence of HRD according to other definitions (as reported above).

The prevalence of any mutation was included regardless of whether it was germline, somatic, or founder. When reported, the details of the mutations were checked to evaluate their pathogenicity or clinical relevance, according to the current guidelines for variant interpretation.^41,42^ Only pathogenic or likely pathogenic or clinically relevant variants were considered for the prevalence analysis, whereas benign, likely benign, and variants of uncertain significance were excluded. If the required information was not reported in the published report, we consulted available online cancer genomic data sets or requested raw data from the authors. In case of missing data, variables were classified as not reported or unclear to avoid misinterpretation. Other definitions of HRD were reported and analyzed separately because of the high level of variability that impeded the performance of proportional meta-analysis.

For each study, data on family history were reviewed by a team member with expertise in hereditary cancer syndromes (R.C.) and every case for which the details were reported was (re-)classified as familial or sporadic according to current National Comprehensive Cancer Network and American College of Medical Genetics and Genomics criteria for genetic cancer risk assessment.^20,43,44^ If family history was not reported, cases were classified as unselected and excluded from the subgroup analysis of sporadic patients to avoid selection bias with potential consequent overestimation of the results.

### Statistical Analysis

#### Meta-analysis.

A random-effect meta-analysis (DerSimonian and Laird model) was performed on the prevalence data to calculate the pooled event rate using the Freeman-Tukey transformation.^45,46^ A Cochran's Q test for heterogeneity was performed reporting the I2 statistic, which indicates the percentage of variation across studies because of heterogeneity rather than chance.^47^ Heterogeneity values of < 30%, 30%-60%, 61%-75%, and > 75% were, respectively, classified as low, moderate, substantial, and considerable.^48^

#### Publication bias and study bias.

Funnel plots of study size against log odds were used to assess publication bias.^49^ The funnel plot asymmetry was assessed by using the Macaskill regression test for binary data.^50^ A general linear (mixed-effects) meta-regression model was also computed, where observations were weighted by the inverse variance of the estimate to allow for heteroscedasticity.^51^

Different strategies for study quality and risk-of-bias appraisal were evaluated.^52^ The available options could be improved for specific application to translational cancer genomic studies and for systematic reviews that incorporate multiple study designs. Risk Of Bias In Non-randomized Studies—of Interventions (ROBINS-I) demonstrated to be the most appropriate and adaptable tool and, as a consequence, was used for these analyses.^53^ In addition, an internal risk-of-bias assessment at the study level was specifically developed: Translating-ROB (ie, TRANSLATIonal caNcer Genomic Risk Of Bias). According to this tool, a 25-point quality rating was applied to each study (Data Supplement).

#### Meta-regression.

A mixed-effects meta-regression analysis was conducted to examine the possibility of effect modification of the pooled prevalence estimates. The mixed-effects meta-regression estimates were computed accounting for the nonlinearity for risk-of-bias score using the restricted cubic spline method.^48^ The following variables were considered possible moderators of the dependent variable: sequencing methodology (non-NGS *v* NGS); stage of disease (early *v* metastatic); risk-of-bias score (Translating-ROB and ROBINS-I); and study sample size (Macaskill test *P* value was applied as indicator of the effect of the study size on outcome). The model estimates were adjusted within genes for the multiplicity of testing using the Benjamini-Hochberg correction.^48^

Mean and standard deviation or median with interquartile range were reported in cases of normally or non-normally distributed data, respectively. Statistical analysis was performed using R (R Foundation for Statistical Computing, Vienna, Austria, v. 4.02)^54^ with the metafor 2.4-0^55^ and FactoMineR packages.^56^

## RESULTS

### General Findings

A total of 2,062 nonduplicate titles and abstracts were retrieved from PubMed, Scopus, and Cochrane databases, and hand-to-hand searches. Three publicly available cancer genomic data sets were consulted.^57-59^ After screening abstracts and titles, 1,918 studies were judged not relevant. After screening full texts, an additional 73 studies that did not meet the eligibility criteria were excluded, with a total of 71 meeting inclusion or exclusion criteria for the systematic review. After a more detailed analysis of the records, 12 manuscripts were further excluded (85 excluded studies are reported in the Data Supplement), and a final total of 59 were included in the systematic review (Data Supplement). Only one cancer genomic data set^57^ was included. The other two were excluded because of overlap with published studies, containing more detailed information.^7,21,60^ In addition, a further three studies included in the systematic review were excluded from the meta-analysis because of selection bias or granularity in data reporting.

**FIG 1.**
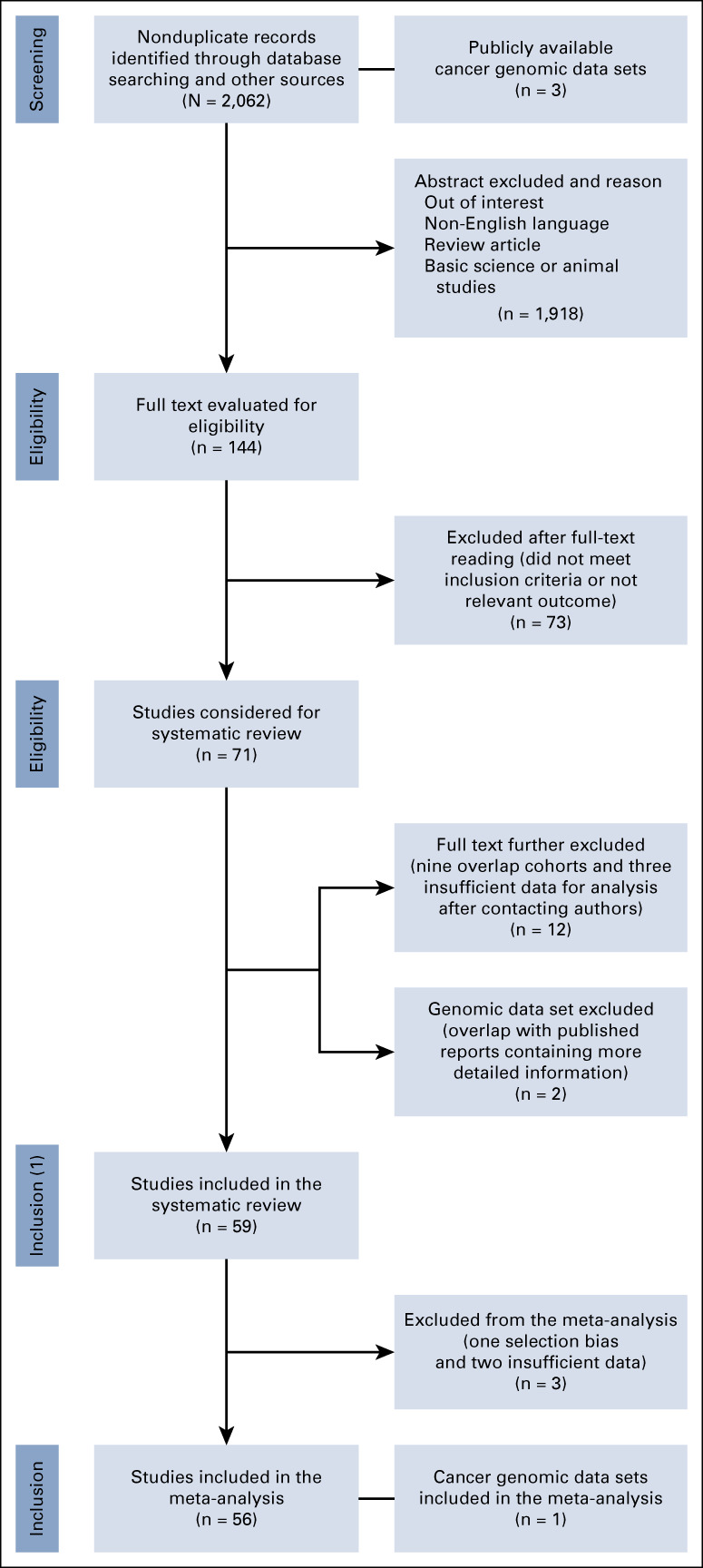
Preferred Reporting Items for Systematic Reviews and Meta-Analyses flowchart of patient selection for the meta-analysis.

Therefore, 59 studies and one cancer genomic data set with 21,842 participants from 18 countries (Data Supplement) met the systematic review's inclusion criteria, whereas 56 studies and the genomic data set were included in the meta-analysis. The flowchart of the study selection process is reported in Figure 1.

The 60 studies (including the data set) included in the systematic review are summarized in the Data Supplement. The majority were from the United States (33 out of 60, 55%). The median number of patients per study was 81 (interquartile range 226). Thirteen studies were conducted in resected patients, seven in metastatic, and one in locally advanced disease, and 16 studies enrolled patients with mixed clinical stage. The remaining studies (23 out of 60, 38%) did not report this information. *BRCA1/2* were the most frequently studied genes (54 out of 60 studies, of which 45 reported information on *BRCA1* and 52 on *BRCA2*), followed by *PALB2* (43 out of 60), *ATM* (35 out of 60), *CHEK2* (30 out of 60), *FANC* genes (27 out of 60), *RAD51* genes (26 out of 60), and *ATR* (21 out of 60). Germline mutations were tested in 54 studies, somatic in 19, and 13 reports included somatic and germline mutations.

Ethnicity was reported in 35 out of 60 (58%) studies (Data Supplement). When reported, Caucasian or White was the most represented ethnicity. Eleven studies (18.3%) that did not specify ethnicity were presumed to be conducted in Caucasian or White populations based on the geographic location of the participating institution. Four studies enrolled Asian patients only. A total of 17 out of 60 (28.3%) studies included patients with AJ ancestry, nine of which reported the population-specific mutation rate and were included in the subgroup analysis.

Several sequencing methodologies had been used, including targeted Sanger sequencing, targeted NGS of individual genes, targeted NGS of multiple genes, targeted capture NGS, WES, and WGS (Data Supplement).

Overall, 13 out of 60 (21.7%) studies enrolled patients with familial PDAC and 27 (45%) included unselected populations but reported details on family history. The remaining 20 (33.3%) included unselected patients and were as a consequence excluded from this specific subgroup meta-analysis (Data Supplement).

### Pooled Prevalence Estimates

Detailed results of the pooled prevalence estimates of mutations in individual HRD genes are reported in Table 1. The pooled proportion of germline and somatic mutations in all included studies was *BRCA1*: 0.9%, *BRCA2*: 3.5%, *PALB2*: 0.2%, *ATM*: 2.2%, *CHEK2*: 0.3%, *FANC*: 0.5%, *RAD51*: 0.0%, and *ATR*: 0.1%. The pooled proportion of germline mutations was *BRCA1*: 0.9% (2.4% in AJ), *BRCA2*: 3.8% (8.2% in AJ), *PALB2*: 0.2%, *ATM*: 2%, *CHEK2*: 0.3%, and *FANC*: 0.4%. No significant differences in the estimates were identified between sporadic and familial cases.

**TABLE 1.**
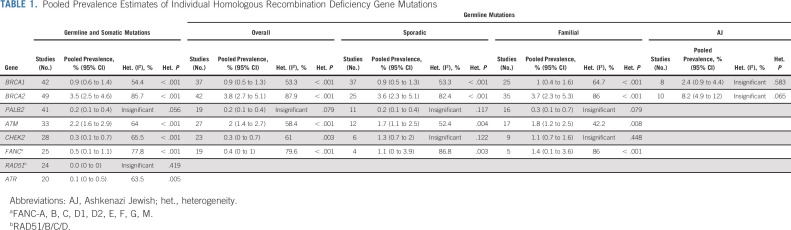
Pooled Prevalence Estimates of Individual Homologous Recombination Deficiency Gene Mutations

For each main outcome, the following tests were performed and reported: the funnel plot of study size against log odds, the forest plot of the prevalence meta-analysis, the linear mixed-effect meta-regression of event rates according to bias score, and event rates according to sample size (Data Supplement). The funnel plots for the prevalence outcomes showed that globally, the event rates for the studies considered fell within the confidence bounds of the plot (low-publication bias zone), indicating an acceptable publication bias result. *BRCA2* was the outcome reporting the greatest number of studies outside the 95% CI.

Meta-regression analysis identified no significant effect modifiers on mutation prevalence for the main end points including sequencing methodology, stage of disease, sample size, and risk-of-bias score at study level (Data Supplement). Detailed biostatistics is reported in the Data Supplement.

The risk of bias according to ROBINS-I and Translating-ROB as well as the validation procedure of Translating-ROB is reported in the Data Supplement. According to ROBINS-I, 45 out of 57 studies (79%) resulted at low or moderate risk of bias.

### HRD Prevalence According to Other Definitions

A total of nine studies reported (1) the prevalence of mutations in additional genes beyond those selected for the meta-analysis; (2) other definitions of HRD (genomic scars, mutational signatures, and structural variation patterns); and (3) only the overall HRD prevalence, without specifying single gene alterations. HRD prevalence ranged between 14.5%-16.5% when extended NGS panels were used and 24%-44% through WGS or WES (Table 2).

**TABLE 2.**
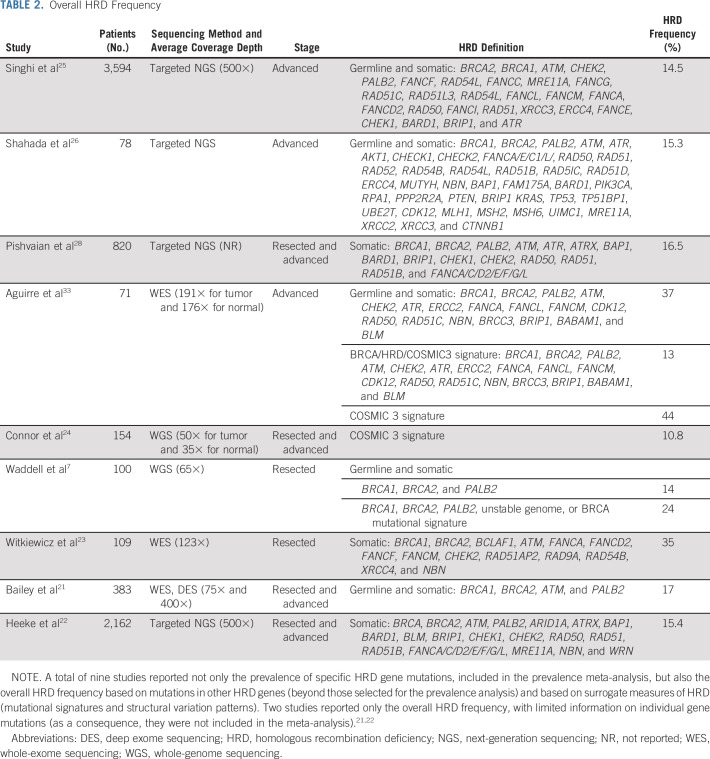
Overall HRD Frequency

The estimated prevalence of the HRD 15-gene list used clinically for metastatic castration-resistant prostate cancer was calculated using an integrated analysis of data from this study and the three online publicly available data sets mentioned in the search strategy. Online data sets provide data only on somatic mutations. Therefore, the latter information was combined with the pooled prevalence estimated of germline mutations in 8 out of 15 overlapping genes from this meta-analysis. The prevalence of the HRD 15-gene list in PDAC was approximately 12% (likely underestimated because of the methodologic limitations in the computation) (Data Supplement).

### LOH (Loss of the Wild-Type Allele) and Somatic Versus Germline Status

Only 10 studies evaluated the somatic event in the second allele. Significant variation in the somatic event (from 0% to 100%) was observed. When only studies with larger cohorts were considered, the overall rate of LOH for all genes tested was approximately 50% (Data Supplement).^24,33,61,62^

Considering the low numbers, the frequency of germline versus somatic mutations was computed for *BRCA1* and *BRCA2* only, with 67% of *BRCA* mutations germline and 33% somatic (Data Supplement).

## DISCUSSION

To our knowledge, this is the first systematic review and meta-analysis assessing the prevalence of HRD in PDAC. There was significant variation in the prevalence of HRD estimates based on current and variable HRD definitions and methods of assessment, with a prevalence of 7.7% using mutation testing of the better characterized HRD genes, 14.5%-16.5% through extended NGS panels, and 24%-44% through WES or WGS. The main contribution to HRD was through *BRCA2*, *BRCA1*, and *ATM*, followed by *FANC* genes, *CHEK2*, and *PALB2*. The prevalence of aberrations in *RAD51*, *ATR*, *BRIP1*, *BARD1*, *CDK12*, and *CHEK1* was markedly less, whereas evidence for rarer genes, such as *RAD54L* and *PPP2R2A*, is lacking. These findings support the increasing interest in *ATM* as it constitutes one of the more common and potentially actionable HRD genes in PDAC.^63-67^

**FIG 2.**
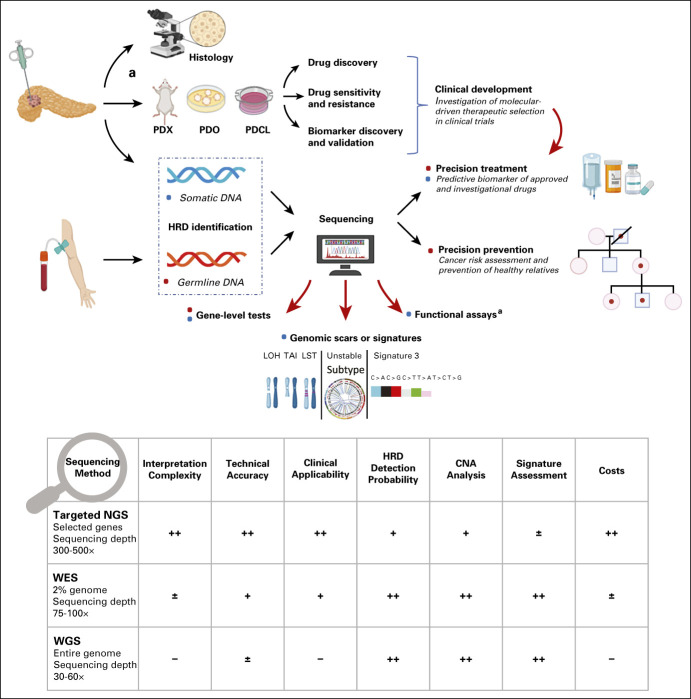
Overview of HRD identification and clinical implications. Although WGS represents the most comprehensive method for HRD identification as it delivers integrated analyses of all genomic events, many barriers limit its utilization in the clinic, feasibility of accessing fresh biopsy material of sufficient size, cost, and analytic complexity. WES is a more accessible strategy and is often proposed as the second choice. However, it seems not to be the optimal method for cancer profiling as many driver events occur outside the coding exome may be missed, on one hand, and the majority of included genes are not cancer genes, on the other. Despite some technical limitations, targeted-capture sequencing delivering comprehensive genomic information, including individual gene mutations, signatures, and structural variation patterns, may represent a reasonable option for real-world applicability (practical and financial advantages compared with WES and WGS). Rating level of sequencing technologies: ++, optimal; +, good; ±, low; –, poor. ^a^Functional assays for real-time HRD status require in vivo or in vitro experiments. CNA, copy-number alterations; HRD, homologous recombination deficiency; LOH, loss of heterozygosity; LST, large-scale transitions; NGS, next-generation sequencing; PDCL, patient-derived cell lines; PDO, patient-derived organoids; PDX, patient-derived xenograft; TAI, telomeric allelic imbalance; WES, whole-exome sequencing; WGS, whole-genome sequencing.

We estimated that the 15-gene list used in prostate cancer may capture approximately 12% of patients with PDAC with HRD tumors. Nonetheless, it is important to underline that although the extension of the genes included in NGS panel testing increases the probability of HRD identification, the clinical relevance of HRD candidates, beyond the core *BRCA1*, *BRCA2*, and *PALB2* genes, has yet to be established.^68,69^ Many other outstanding questions still need to be addressed, including the clinical differences between somatic versus germline mutations, monoallelic versus biallelic inactivation (including epigenetic silencing), the specific functional consequence of each molecular alteration in predicting therapeutic sensitivity to therapy with platinum, PARPi, and other novel agents that target DDR, and the mechanisms of primary or secondary resistance (including the role of secondary mutations in DDR genes). A possible solution to assessing the functional contribution of each specific alteration, or potentially biomarkers in their own right, is to assess various patterns in genomic aberrations across the genome that represent defects in DDR mechanisms. These surrogate genomic readouts of HRD such as genomic scarring as well as point mutational and structural variant signatures^70-72^ have the potential to deliver clinically relevant genomic information and to detect HRD beyond point mutations in known HR genes in an additional 10%-15% of tumors in cohort-based and preclinical studies.^7,36,70,73^ Our results showed that when these surrogate measures are used, like in studies based on WGS technologies, the probability of capturing HRD rises significantly (up to 44%).^7,33^ The central challenge is that although WGS can define putative biomarkers of therapeutic response in cohort studies of breast^73^ and pancreatic cancer,^19^ these methodologies are not currently translatable to the clinic. Routine formalin-fixed clinical biopsies used for sequencing are often small, and current diagnostic assays are mostly focused on the coding regions. Technology continues to advance, and one day, WGS may integrate seamlessly into the health system and deliver routine results; however, in the meantime, we require a feasible diagnostic that can capture surrogate readouts of HRD that can be tested in clinical trials. An additional current challenge is the probability of loss of the second allele in a given HRD gene. Although this occurs in 90% or more in the case of germline *BRCA1* and *BRCA2*,^74,75^ allowing the assumption that if a mutation in one allele is detected, the second is inactivated in 90%, the rate of loss of the second allele for somatic *BRCA* mutations is not well characterized. In particular, germline and somatic events in other HRD genes are largely undefined. The second allele is often inactivated through copy-number alterations and structural variants and can be difficult to detect and interpret, especially if the epithelial cellular content falls below 30%, with rates of epigenetic inactivation largely unknown beyond *BRCA*. This different rate of second allelic loss impacts on penetrance for predisposition assessments of novel candidate genes and substantially affects therapeutic development for non-*BRCA* HRD genes. HRD driven by gene-level events beyond the core HRD genes may potentially be the consequence of a large diverse group of genes with low rates of second allelic inactivation, making surrogate measures more attractive. Setting a threshold to define HRD and the predictive therapeutic value of HRD defined in this way as with noncore HRD genes will require clinical testing and validation.^7,26,33,76,77^ What is needed is a feasible diagnostic that can assess gene-level events and signatures and can use formalin-exposed material from small biopsies (Fig 2).^78,79^ Park et al^80^ recently showed that pathogenic somatic or germline mutations in core HRD genes (*BRCA1/2*, *PALB2*) and biallelic loss of other rarer HRD genes determined through targeted-capture NGS are associated with improved survival in patients with advanced HRD PDAC treated with platinum-based chemotherapy. Loss of the second allele was more prevalent in *BRCA1* and *BRCA2* versus other HR genes. They were also able to determine surrogate measures of HRD in a subgroup of patients (large-scale transition, point mutational Signature 3,^70^ and genomic instability) from the same assay, which predicted platinum response and improved survival. Notably, HRD PDAC is associated with increased tumor mutation burden,^81^ offering opportunities for combining immunotherapy with PARP inhibition in this subgroup.^80,82^

Concerning germline alterations, our results further support the current National Comprehensive Cancer Network guidelines recommending routine screening for germline variants in patients with PDAC at diagnosis, regardless of age, ancestry, and family or personal history of cancer, including not only *BRCA1/2* but also *ATM*, *CDKN2A*, *PALB2*, *STK11*, *TP53*, *MLH1*, *MSH2*, *MSH6*, and *PMS2*.^20^ Based on our findings, *FANC* genes and *CHEK2* should be added to that list. Given the relevant clinical implications of identifying mutation carriers without a family history, current guidelines for germline testing should be reassessed.^62,83,84^ Broader testing would maximize the identification of patients suitable for approved and investigational therapies and would positively impact on prevention strategies of a broad spectrum of tumors in healthy family members through cascade testing.^13,85,86^

The result of this systematic review and meta-analysis should be interpreted with caution, given the following limitations: (1) ascertainment bias because of the exclusion of manuscripts that were not considered of interest because of vague methods or reporting of results; (2) limited data on baseline patient characteristics in the majority of studies; (3) heterogeneity among studies and included patient populations; (4) variability in sequencing methodologies; (5) considerable heterogeneity in some meta-analysis results; (6) inherent publication bias, where research on the topic may be skewed toward the publication of significant results only^47,87^; and (7) risk of bias at study level. In this regard, there was significant inaccuracy and inconsistency in reporting methods and results, thus making conclusions partially comparable only. This, together with the aforementioned limitations, likely hampers analysis and interpretation. To improve study quality and ensure transparency and standardization of reporting results, we propose a modified (m-) REMARK criteria^88^ as a checklist for future translational cancer genomic studies (Table 3). m-REMARK has also been used to derive an internal risk-of-bias assessment tool at study level, specifically developed for studies focused on germline or somatic mutation analysis. After this initial validation, further investigation will be essential to more accurately validate Translating-ROB, as well as m-REMARK.

**TABLE 3.**
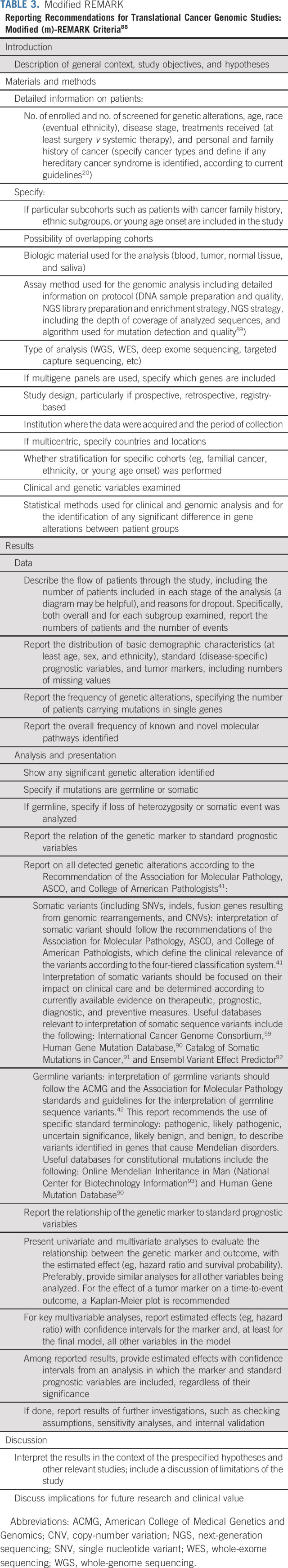
Modified REMARK

Because of general poor reporting of information, it was not possible to systematically assess prevalence variations according to disease stage or patient age. However, individual studies did not report substantial discrepancies in HRD frequency between early-stage and late-stage patients.^8,28,94^ Similarly, the majority of studies did not identify statistically significant age variations between germline mutation carriers and wild-type patients,^8,61,95-98^ and three studies evaluating the mutation rate in young-onset patients (< 50 years) did not find a significant difference compared with older patients.^25,83,99^ Further large studies are necessary to clarify these important aspects.

Last, general poor reporting of ethnicity and focus on Caucasian or White populations of the majority of studies not only represents a limitation that hampers the wide generalizability of research findings to underrepresented populations, but also highlights major disparities in access to cancer research programs. Interestingly, geographic and ethnic heterogeneity of *BRCA* mutation prevalence among patients with PDAC has recently been described,^100^ but further studies are needed to understand ethnic variations of genomic events and related clinical implications.

In conclusion, HRD constitutes a prevalent and clinically relevant pathway in PDAC. Preclinical and clinical data support that every patient with newly diagnosed PDAC should be tested for HRD and ideally enrolled in biomarker-enriched clinical trials (Table 4). Based on our study and available literature, integrated HRD assessment, including germline and somatic analysis, represents the current ideal approach, with the highest potential to drive therapeutic choices not only in metastatic but also in early-stage disease.^13,80,85,86,101^ Nevertheless, major efforts are necessary to harmonize HRD definition and to find the optimal biomarker for treatment selection. Although surrogate readouts of HRD can identify a greater proportion of patients with HRD than analyses limited to gene-level approaches, they need to be assessed in clinical trials, and before widespread adoption would require a diagnostic capable of feasibly detecting genomic signatures in the majority of patients. Expanding research on integrated WGS or WES and transcriptomic profiling, together with functional analyses, is also necessary to unravel the complex biology of HRD in PDAC, to elucidate the predictive value of HRD aberrations beyond core genes, and to understand the real-time HRD status.

**TABLE 4.**
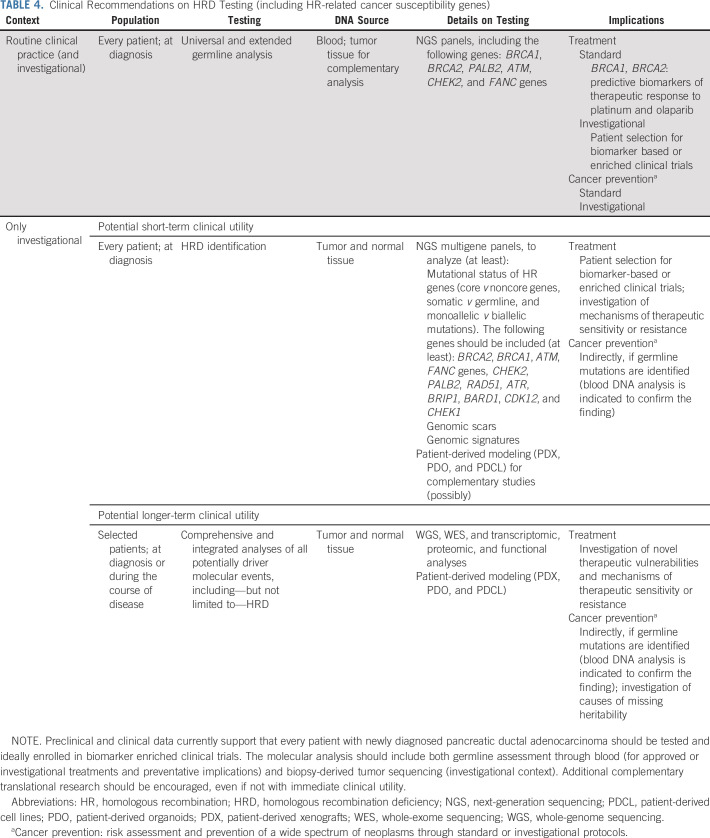
Clinical Recommendations on HRD Testing (including HR-related cancer susceptibility genes)
